# Supplementary material for: Differentiation and Integration of Competing Memories: A Neural Network Model
Source: bioRxiv. 2024 Jun 25:2023.04.02.535239. Preprint. [Version 4] doi: 10.1101/2023.04.02.535239 (PMC10103961; doi:10.1101/2023.04.02.535239)
Supplement: Supplement 1 [file NIHPP2023.04.02.535239v4-supplement-1.pdf]

bioRxiv preprint doi: https://doi.org/10.1101/2023.04.02.535239; this version posted June 25, 2024. The copyright holder for this preprint (which was not certified by peer review) is the author/funder, who has granted bioRxiv a license to display the preprint in perpetuity. It is made available under aCC-BY 4.0 International license.

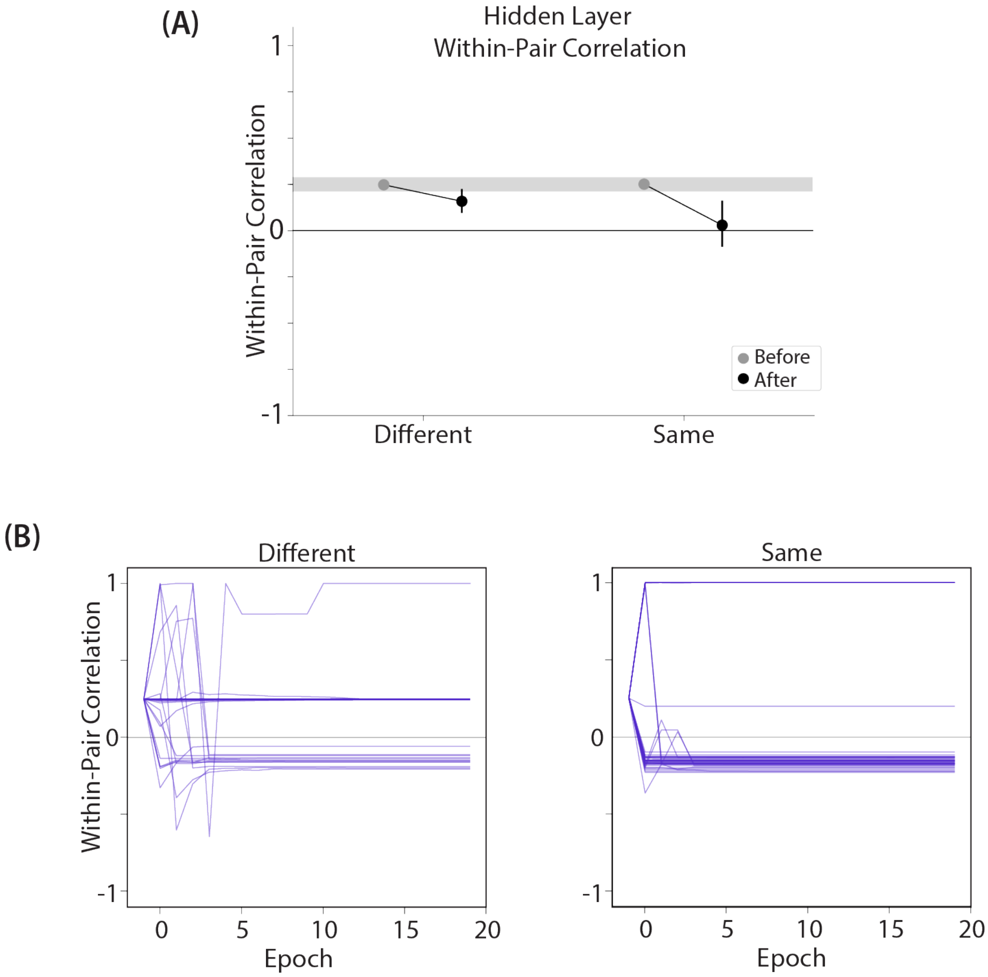

Figure 8–Figure supplement 1. Results from our model of ***Favila et al.*** (***2016***), using an alternative parameterization where the oscillation amplitude *Osc* for the hidden layer is set to 0.1 instead of 0.067: (A) Within-pair correlation between A and B hidden layer representations before and after learning. Error bars indicate the 95% confidence interval around the mean. Compare with ***Figure 8***A. When *Osc* is set to 0.1, both the different-face and same-face conditions show a reduction in pattern similarity compared to baseline, but the size of this decrease is larger in the same-face condition. This pattern of results qualitatively aligns with the actual results observed by ***Favila et al.*** (***2016***). (B) Plots of the within-pair correlation across learning epochs, shown separately for the different-face and same-face conditions. Each purple line is a separate run of the model (darker purple lines indicate many lines superimposed on top of each other). The plots show that individual model runs exhibit one of three discrete outcomes (integration, reflected by a within-pair correlation of 1; no change; or differentiation, reflected by a negative within-pair correlation). The differences in average levels of representational change shown in part (A) for the same-face and different-face conditions are due to differences in the frequencies-of-occurrence of these three discrete outcomes.

bioRxiv preprint doi: https://doi.org/10.1101/2023.04.02.535239; this version posted June 25, 2024. The copyright holder for this preprint (which was not certified by peer review) is the author/funder, who has granted bioRxiv a license to display the preprint in perpetuity. It is made available under aCC-BY 4.0 International license.

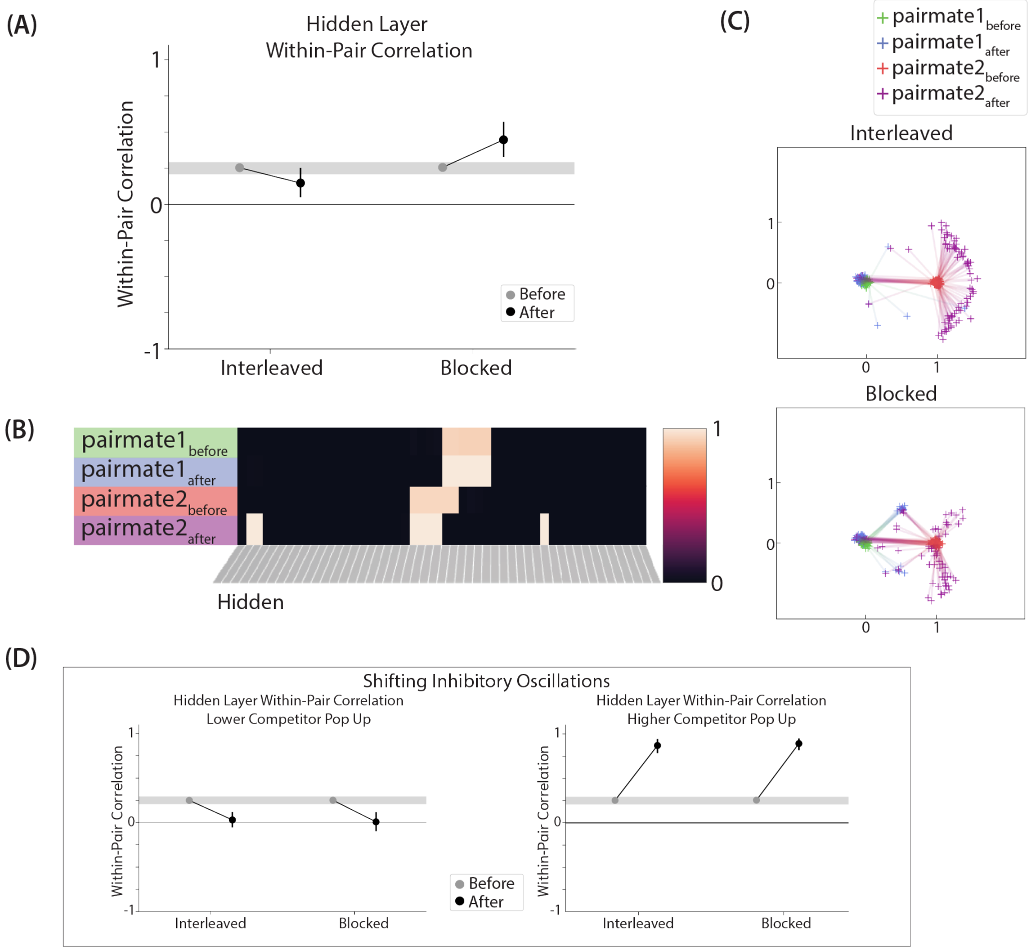

1494

Figure 10–Figure supplement 1. Results from our model of ***Schlichting et al.*** (***2015***), using an alternative parameterization where the connection strength between X (in the output layer) and A (in the hidden layer) is set to .9 in the blocked condition (instead of .999). (A) Within-pair correlation between hidden-layer A and B representations is shown before and after learning; here, the oscillation amplitude *Osc* was set to .0635. In the interleaved condition, the within-pair correlation is reduced after learning, indicating differentiation. In the blocked condition, the within-pair correlation increases, indicating integration. (B) Activity patterns of both pairmates in the hidden layer before and after learning are shown for a sample run in the interleaved condition. Asymmetry in distortion can be seen in how pairmate 2, but not pairmate 1, picks up additional units that did not previously belong to either representation. (C) MDS plots for each condition illustrate the pattern of representational change in the hidden layer. In the blocked condition, the pairmates integrate and move toward each other on most (but not all) trials — a subset of the trials show differentiation. In the interleaved condition, differentiation occurs on most (but not all) trials — a subset of the trials show integration. (D) To investigate how these results might vary across brain regions with different inhibitory dynamics, we manipulated the inhibitory oscillation amplitude to change the amount of competitor pop-up. No parameters other than the inhibitory oscillation amplitude were changed. When oscillation amplitude is reduced to .0615, less competitor pop-up happens, and here this results in both conditions showing differentiation (compare to our original results in ***Figure 10***D, where differentiation occurred in the blocked condition but not the interleaved condition). When oscillation amplitude is raised to .07, more competitor pop-up happens, and both conditions lead to integration. For panels A and D, error bars indicate the 95% confidence intervals around the mean.
